# Species Tree Estimation and the Impact of Gene Loss Following Whole-Genome Duplication

**DOI:** 10.1093/sysbio/syac040

**Published:** 2022-06-11

**Authors:** Haifeng Xiong, Danying Wang, Chen Shao, Xuchen Yang, Jialin Yang, Tao Ma, Charles C Davis, Liang Liu, Zhenxiang Xi

**Affiliations:** Key Laboratory of Bio-Resource and Eco-Environment of Ministry of Education, College of Life Sciences, Sichuan University, Chengdu 610065, China; Key Laboratory of Bio-Resource and Eco-Environment of Ministry of Education, College of Life Sciences, Sichuan University, Chengdu 610065, China; Key Laboratory of Bio-Resource and Eco-Environment of Ministry of Education, College of Life Sciences, Sichuan University, Chengdu 610065, China; Key Laboratory of Bio-Resource and Eco-Environment of Ministry of Education, College of Life Sciences, Sichuan University, Chengdu 610065, China; Department of Statistics and Institute of Bioinformatics, University of Georgia, Athens, GA 30602, USA; Key Laboratory of Bio-Resource and Eco-Environment of Ministry of Education, College of Life Sciences, Sichuan University, Chengdu 610065, China; Department of Organismic and Evolutionary Biology, Harvard University Herbaria, Cambridge, MA 02138, USA; Department of Statistics and Institute of Bioinformatics, University of Georgia, Athens, GA 30602, USA; Key Laboratory of Bio-Resource and Eco-Environment of Ministry of Education, College of Life Sciences, Sichuan University, Chengdu 610065, China

## Abstract

Whole-genome duplication (WGD) occurs broadly and repeatedly across the history of eukaryotes and is recognized as a prominent evolutionary force, especially in plants. Immediately following WGD, most genes are present in two copies as paralogs. Due to this redundancy, one copy of a paralog pair commonly undergoes pseudogenization and is eventually lost. When speciation occurs shortly after WGD; however, differential loss of paralogs may lead to spurious phylogenetic inference resulting from the inclusion of pseudoorthologs–paralogous genes mistakenly identified as orthologs because they are present in single copies within each sampled species. The influence and impact of including pseudoorthologs versus true orthologs as a result of gene extinction (or incomplete laboratory sampling) are only recently gaining empirical attention in the phylogenomics community. Moreover, few studies have yet to investigate this phenomenon in an explicit coalescent framework. Here, using mathematical models, numerous simulated data sets, and two newly assembled empirical data sets, we assess the effect of pseudoorthologs on species tree estimation under varying degrees of incomplete lineage sorting (ILS) and differential gene loss scenarios following WGD. When gene loss occurs along the terminal branches of the species tree, alignment-based (BPP) and gene-tree-based (ASTRAL, MP-EST, and STAR) coalescent methods are adversely affected as the degree of ILS increases. This can be greatly improved by sampling a sufficiently large number of genes. Under the same circumstances, however, concatenation methods consistently estimate incorrect species trees as the number of genes increases. Additionally, pseudoorthologs can greatly mislead species tree inference when gene loss occurs along the internal branches of the species tree. Here, both coalescent and concatenation methods yield inconsistent results. These results underscore the importance of understanding the influence of pseudoorthologs in the phylogenomics era. [Coalescent method; concatenation method; incomplete lineage sorting; pseudoorthologs; single-copy gene; whole-genome duplication.]

The vast increase in genomic data has revealed that whole-genome duplication (WGD) or polyploidy is widespread (Van de Peer et al. 2017) and has been demonstrated in diverse taxa, including ciliates (Aury et al. 2006), yeasts (Marcet-Houben and Gabaldón 2015; Wolfe 2015), horseshoe crabs (Kenny et al. 2016), hexapods (Li et al. 2018), teleosts (Glasauer and Neuhauss 2014; Lien et al. 2016), amphibians (Session et al. 2016), and especially plants (Clark and Donoghue 2018; Leebens-Mack et al. 2019; Cai et al. 2019). Some estimates suggest that one-half to two-thirds of flowering plants are polyploid (Rieseberg and Willis 2007; Moghe and Shiu 2014; Soltis et al. 2015), and an astonishingly diverse range of lineages show evidence of WGD. For instance, eight WGDs have been identified in Brassicaceae (Kagale et al. 2014), at least 22 ancient WGDs have been inferred for Malpighiales (Cai et al. 2019), 26 ancient and more recent WGDs have been demonstrated in Caryophyllales (Yang et al. 2018), and 34 WGDs have been recorded in Andropogoneae (Estep et al. 2014).

Immediately following WGD, most genes are present in two copies as paralogs (i.e., paralogs or sometimes referred to as ohnologs to honor Ohno and his contribution to this area; Ohno 1970). Due to this redundancy, one copy of a paralog pair often undergoes pseudogenization and is eventually lost (Lynch and Conery 2000; Langham et al. 2004; Aury et al. 2006; Makino and McLysaght 2012). The longevity of a duplicate gene can be quantified by its half-life (i.e., the amount of time for half of the duplicates derived from a single WGD to be lost; Panchy et al. 2016), and the approximate half-lives for gene duplicates in *Arabidopsis*, humans, fruit flies, nematodes, and fungi are 17.3, 7.5, 3.2, 1.7, and 1.0 myr, respectively (Lynch and Conery 2003). In addition, a recent comparative genomics study inferred the evolutionary histories of nearly 7000 protein-coding genes following the teleost-specific WGD ($\sim$306 Ma), and identified that more than 70–80$\%$ of duplicated genes in nine teleosts were lost in the first 60 myr (Inoue et al. 2015).

Extensive loss of paralogs may greatly impact phylogenomic inference, which relies heavily on data sets that comprise single-copy orthologous genes, that is, genes that result from speciation rather than arising from gene duplication (Creevey et al. 2011; Hellmuth et al. 2015; Li et al. 2017). In recent years, researchers have been particularly concerned about the negative impacts of pseudoorthologs on phylogenomic inference (Smith and Hahn 2022; sometimes referred to as out-paralogs; Sonnhammer and Koonin 2002)—pseudoorthologs are paralogous genes mistakenly identified as orthologs because they are present in single copies within each sampled species. In short, gene trees inferred from pseudoorthologs may differ greatly from the species tree. Along these lines, if speciation occurs shortly after WGD and subsequent loss of paralogs is restricted to one major paralog subclade, single-copy genes should include only one-to-one orthologs and be relatively straightforward to analyze phylogenetically (Fig. 1a). In contrast, when both copies of a paralog pair within post-WGD species are equally likely to be lost, paralogous gene copies may be erroneously grouped as orthologs (i.e., pseudoorthologs) and lead to incorrect gene tree estimation (Fig. 1b) (Salichos and Rokas 2011; Struck 2013; Smith and Hahn 2022). This is particularly relevant in plants, which are prone to rampant WGD (De Smet et al. 2013; Hollister 2015). A recent study has shown that on average 64.5$\%$ of plant genes are paralogous, ranging from 45.5$\%$ in the bryophyte *Physcomitrella* *patens* to 84.4$\%$ in apple (Panchy et al. 2016). Furthermore, the occurrence of positionally biased and lineage-specific losses of paralogs has been demonstrated in eukaryotic genomes (Postlethwait 2007; Makino and McLysaght 2012; Campbell et al. 2019), which could further complicate the identification of pseudoorthologs, thus affecting phylogenomic inference.

**Figure 1.**
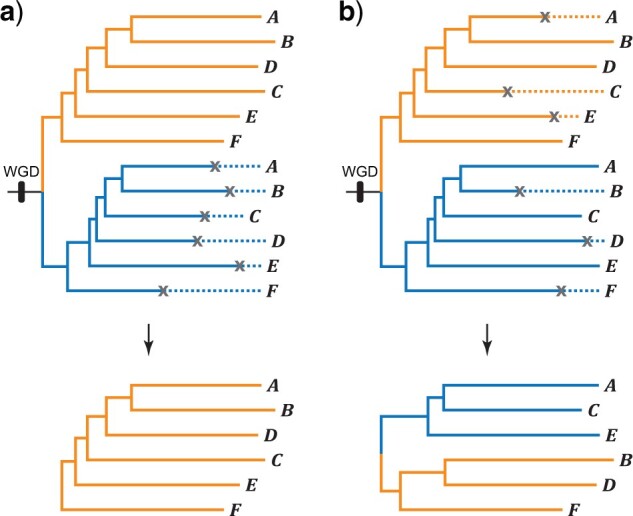
Two possible scenarios for the loss of paralogs following speciation. Two major subclades of paralogs originate from WCD (or just duplication). a) Loss of paralogs is restricted to one major subclade of paralogs, resulting in a single-copy gene includes only orthologs. b) Both copies of a paralog pair within post-WGD species are equally likely to be lost and paralogs may be erroneously grouped as orthologs.

Despite theoretical and methodological advancements in phylogenomics during the last two decades (Bravo et al. 2019), few studies have explicitly examined the impact of paralogs on phylogenomic analyses using empirical data in an explicit coalescent framework. Struck (2013) analyzed a supermatrix of annelids and demonstrated that the placement of taxa with high bootstrap support could be attributed to paralogs. Siu-Ting et al. (2019) assembled a phylogenomic data set for the Lissamphibia using transcriptomic data and identified that paralogs may mislead species tree estimation resulting in spurious relationships. In contrast, two recent studies (Smith and Hahn 2022; Yan et al. 2022) suggest that species tree inference in the presence of paralogs is as accurate as phylogenetic analyses using orthologs. Despite these findings, there lacks a more rigorous assessment of how pseudoorthologs influenced by differential gene loss of paralogous gene copies affect species tree estimation, especially using mathematical models and simulated data. This also presents an opportunity to explore the relative utility of applying more standard concatenation methods versus coalescent methods, which more explicitly model gene tree species tree differences. Concatenation methods (i.e., the maximum likelihood tree inferred from the concatenated sequences across loci) have been commonly employed for species tree estimation, which implicitly assumes that all genes have the same or very similar evolutionary histories. Coalescent-based methods, in contrast, permit gene trees to have different evolutionary histories (Liu et al. 2009a). Some of these methods, including *BEAST (Heled and Drummond 2010), BEST (Liu 2008), and BPP (Flouri et al. 2018), simultaneously estimate gene trees and the species tree from multilocus sequence data. These alignment-based methods have outstanding accuracy, but they are computationally intensive (Leaché and Rannala 2011; Bayzid and Warnow 2013; Mirarab et al. 2016). Other coalescent-based methods infer the species tree from a set of gene trees using likelihood functions, for example, MP-EST (Liu and Yu 2010), STELLS (Wu 2012; Pei and Wu 2017), and STEM (Kubatko et al. 2009). In addition, recently developed methods, including ASTRAL (Mirarab et al. 2014; Mirarab and Warnow 2015; Zhang et al. 2018), STAR (Liu et al. 2009b), and STEAC (Liu et al. 2009b), estimate the species tree from gene trees using summary statistics. However, none of these coalescent methods have measured the effect of pseudoorthologs on species tree estimation to our knowledge. Although the latter consensus methods are not strictly coalescent-based, they can accommodate gene tree discordance due to incomplete lineage sorting (ILS), and have been shown to be statistically consistent under the multispecies coalescent model (MSC) as long as gene tree estimation is not biased (Liu et al. 2009b; Liu and Yu 2011; Mirarab et al. 2014; Xi et al. 2015). A recent study indicates that gene-tree-based methods may be statistically inconsistent in the presence of long-branch attraction when the number of sites is restricted (Roch et al. 2019). For simplicity, we also refer to these methods as gene-tree-based coalescent methods.

Here, using mathematical models, numerous simulated data sets, and two newly assembled empirical data sets, we assess how pseudoorthologs arising from extensive and differential loss of paralogs affect species tree estimation. We focus our efforts on a comparison of coalescent and concatenation methods under varying levels of ILS and differential patterns of gene loss following WGD. Furthermore, we seek to explore how species tree estimation methods are affected by the inclusion of pseudoorthologs in single-copy genes.

## Materials and Methods

### Applying Mathematical Models to Evaluate the Impact of Pseudoorthologs in Single-Gene Assessments of Phylogeny

For simplicity, we assume that a WGD occurs in the ancestral population at the root of a 4-taxon species tree $\boldsymbol{{S}}$, and the topology of the species tree is pectinate $(((A,B),C),D)$ (Fig. S1a of the Supplementary material available on Dryad at http://dx.doi.org/10.5061/dryad.prr4xgxmr) or symmetric $((A,B),(C,D))$ (Fig. S2a of the Supplementary material available on Dryad). For each gene, paralogs evolve along the underlying species tree and diverge when speciation occurs. As a result, gene trees produced by the species tree $\boldsymbol{{S}}$ comprise two major subclades of paralogs, and each subclade includes exactly one copy from each of the species $A$ to $D$, that is, they exist as orthologs within each paralog subclade (Figs. S1b and S2b of the Supplementary material available on Dryad). The genealogical history of eight gene copies $\{A_1,B_1,C_1,D_1,A_2,B_2,C_2,D_2\}$ is a coalescent gene tree generated from the 8-taxon tree (Figs. S1b and S2b of the Supplementary material available on Dryad) under the MSC. Meanwhile, we assume that one of two gene copies within post-WGD species is lost at a rate $\lambda_i$ in the branch $i(i=1,\ldots,7)$ of the species tree (Figs. S1a and S2a of the Supplementary material available on Dryad). Since the waiting time until the next gene loss is an exponential random variable with rate $\lambda_i$, the probability that a gene copy is lost in the branch $i$ is $1-e^{-\tau_i}$ for $i=1,\ldots,7$, where $\tau_i=\lambda_it_i$ and $t_i$ is the length of the branch $i$ (Figs. S1a and S2a of the Supplementary material available on Dryad). If gene loss has occurred in the branches of the species tree, the genealogical tree of retained gene copies follows a coalescent process in a subtree of the 8-taxon tree obtained by pruning the branches leading to the lost copies. For example, if a gene copy (i.e., the red copy in Figs. S1a and S2a of the Supplementary material available on Dryad) is lost at the root of the species tree, the genealogical tree of retained copies $\{A_2,B_2,C_2,D_2\}$ follows a coalescent process along the lineages of the subtree $\boldsymbol{{T}}_{\bf 1}$ (Figs. S1c and S2c of the Supplementary material available on Dryad) obtained by pruning the branches of the lost copies $\{A_1,B_1,C_1,D_1\}$ in the 8-taxon tree (Figs. S1b and S2b of the Supplementary material available on Dryad). Various combinations of gene loss in the branches of the pectinate 4-taxon species tree may result in eight subtrees, that is, $\boldsymbol{{T}}_{\bf 1}=(((A,B),C),D), \boldsymbol{{T}}_{\bf 2} \ = \ (((A,B),C),D), \boldsymbol{{T}}_{\bf 3} \ = \ (((A,B), D),C)$, $\boldsymbol{{T}}_{\bf 4}=(((A,C),D),B), \boldsymbol{{T}}_{\bf 5}=(((B,C),D),A)$, $\boldsymbol{{T}}_{\bf 6}=((A,B)$, $(C$, $D))$, $\boldsymbol{{T}}_{\bf 7}=((A,C),(B,D)),$ and $ \boldsymbol{{T}}_{\bf 8}=((A,D)$, $(B,C))$ (Fig. S1c of the Supplementary material available on Dryad). The subtree $\boldsymbol{{T}}_{\bf 1}$ is identical with the species tree $\boldsymbol{{S}}$, and $\boldsymbol{{T}}_{\bf 2}$ is also topologically identical with the species tree $\boldsymbol{{S}}$ but possesses longer internal branches. Similarly, gene loss in the branches of the symmetric 4-taxon species tree may result in another set of eight subtrees (Fig. S2c of the Supplementary material available on Dryad). Given the subtree $\boldsymbol{{T}}_{\boldsymbol{{i}}}$, the genealogical tree of retained gene copies follows a coalescent probability distribution $P(G|\boldsymbol{{T}}_{\boldsymbol{{i}}},\theta)$ (see Supplementary Material A available on Dryad). Moreover, the probability distribution $P(\boldsymbol{{T}}_{\boldsymbol{{i}}}|S,\lambda)$ of the subtree $\boldsymbol{{T}}_{\boldsymbol{{i}}}$ given the species tree $\boldsymbol{{S}}$ and loss rates $\lambda$ can be derived from the loss process along the lineages of the species tree (see Supplementary Material B available on Dryad). Thus, the probability distribution $P(\boldsymbol{{G}}|\boldsymbol{{S}},\theta,\lambda)$ of a gene tree $\boldsymbol{{G}}$ given the 4-taxon species tree ***S*** (topology and branch lengths), the population size parameters $\theta$, and the loss rates $\lambda=\{\lambda_1\ldots,\lambda_7\}$ is given by

(1) \begin{align*}\label{eq1} P(\boldsymbol{{G}}|\boldsymbol{{S}},\theta,\lambda)= \sum_{i=1}^{8}P(\boldsymbol{{G}}|\boldsymbol{{T}}_{\boldsymbol{{i}}},\theta) P(\boldsymbol{{T}}_{\boldsymbol{{i}}}|S,\lambda). \end{align*}

Next, we consider the probability distribution $P(\boldsymbol{{G}}|\boldsymbol{{S}},\theta,\lambda$) of a gene tree $\boldsymbol{{G}}$ under three scenarios of gene loss, namely, gene loss at the root of the species, gene loss in the internal branches of the species tree, and gene loss in the terminal branches of the species tree.

*Scenario 1: Gene loss at the root of the species tree* (Supplementary Material C available on Dryad). If gene loss occurs at the root of the species tree (i.e., $\tau_1\to\infty$), retained gene copies are exclusively orthologs and the corresponding subtree is $\boldsymbol{{T}}_{\bf 1}$ with probability 1. The genealogical history of retained gene copies follows the coalescent distribution of gene trees given the species tree $\boldsymbol{{S}}$, that is,

(2) \begin{align*}\label{eq2} &P(\boldsymbol{{G}}|\boldsymbol{{S}},\theta,\lambda)\nonumber\\ &\quad{} = \sum_{i = 1}^8 P(\boldsymbol{{G}}| \boldsymbol{{T}}_{\boldsymbol{{i}}}, \theta)P(\boldsymbol{{T}}_{\boldsymbol{{i}}}\vert S,\lambda ) = P(\boldsymbol{{G}}|\boldsymbol{{T}}_{\boldsymbol{{i}}},\theta) = P(\boldsymbol{{G}}|\boldsymbol{{S}},\theta). \end{align*}

This result can be generalized to a species tree of more than four taxa (Supplementary Material C available on Dryad). Therefore, if gene loss occurs at the root of a species tree, the coalescent methods for species tree estimation can consistently estimate the species tree $\boldsymbol{{S}}$ as the number of loci increases. In contrast, concatenation methods are statistically inconsistent if the species tree $\boldsymbol{{S}}$ is in the anomaly zone.

*Scenario 2: Gene loss among the internal branches of the species tree* (Supplementary Material D available on Dryad). Previous studies have demonstrated that the occurrence of gene loss among the internal branches of the species tree (i.e., reciprocal gene loss following WGD) may result in pseudoorthologs (Scannell et al. 2006, 2007; Sémon and Wolfe 2007; Maclean and Greig 2011). For example, if gene loss occurs in the internal branch 3 of the pectinate 4-taxon species tree (Fig. S1a of the Supplementary material available on Dryad), the probability of the subtree $\boldsymbol{{T}}_{\bf 6}$ converges to 1 (see Scenario 2 in Supplementary Material D available on Dryad). Since the probability distribution of a gene tree $\boldsymbol{{G}}$ for pseudoorthologs is dominated by the coalescent distribution of a gene tree $\boldsymbol{{G}}$ generated from the subtree $\boldsymbol{{T}}_{\bf 6}$, coalescent methods consistently recover the subtree $\boldsymbol{{T}}_{\bf 6}$, which is incongruent with the species tree $\boldsymbol{{S}}$. Therefore, both coalescent and concatenation methods are statistically inconsistent under these circumstances.

*Scenario 3: Gene loss in the terminal branches of the species tree* (Supplementary Material E available on Dryad). If gene loss occurs in the terminal branches or the misidentification rates of paralog/ortholog programs are independent of each other across species, it indicates that gene copies are independently retained for species $A$ to $D$. Let $p_x$ be the probability that the gene copy (i.e., the red copy in Figs. S1a and S2a of the Supplementary material available on Dryad) within the first major subclade of paralogs is lost for the species $x$ (where $x = A, B, C, D)$, and $(1 - p_x )$ is the probability that the gene copy (i.e., the green copy in Fig. S1a of the Supplementary material available on Dryad) within the second major subclade is lost for species $x$. We first consider the case that gene copies $[A_1,B_1,C_1,D_1 ]$ from the first major subclade or $[A_2,B_2,C_2,D_2 ]$ from the second major subclade are retained in the terminal branches of the species tree, that is, the four probabilities $p_x$ are equal to 1 or 0 (i.e., $p_A = p_B = p_C = p_D = 1$ or $p_A = p_B = p_C = p_D = 0)$. The probability distribution of the subtree $\boldsymbol{{T}}$ becomes $P\left({\boldsymbol{{T}}_{\bf 1} | \boldsymbol{{S}},\lambda} \right) = 1$ and $P\left( {\boldsymbol{{T}}_{\boldsymbol{{i}}} | \boldsymbol{{S}},\lambda} \right) = 0$ for $i = 2,\ldots,8$, leading to Scenario 1 described above. If $(p_A = p_B = 1,p_C = p_D = 0)$ or $(p_A = p_B = 0,p_C = p_D = 1)$, the probability distribution of the subtree $\boldsymbol{{T}}$ becomes $P\left( {\boldsymbol{{T}}_{\bf 6} | S,\lambda} \right) = 1$ and $P\left( {\boldsymbol{{T}}_{\boldsymbol{{i}}} | \boldsymbol{{S}},\lambda} \right) = 0$ for $i \ne 6$, leading to Scenario 2 described above. If two gene copies ($A_1$ and $A_2$, $B_1$ and $B_2$, $C_1$ and $C_2$, $D_1$ and $D_2 )$ in the terminal branches of the species tree are equally likely to be lost (i.e., $p_A = p_B = p_C = p_D = 0.5)$, the eight subtrees for the pectinate or symmetric 4-taxon species tree are uniformly distributed with probabilities $P\left( {\boldsymbol{{T}}_{\boldsymbol{{i}}} | \boldsymbol{{S}},\lambda} \right) = \frac{1}{8}$ for $i = 1,\ldots,8$ (Supplementary Material E.3 available on Dryad). In the presence of ILS, each of the eight subtrees may possess different gene trees, and the probability distribution $P(\boldsymbol{{G}}| \boldsymbol{{T}}_{\boldsymbol{{i}}},\theta )$ of a gene tree $\boldsymbol{{G}}$ given the subtree $\boldsymbol{{T}}_{\boldsymbol{{i}}} \left( {i = 1, 2,\ldots, 8} \right)$ can be derived from the coalescent theory. If the lengths of internal branches of the species tree $\boldsymbol{{S}}$ approach zero (in coalescent units; i.e., a high level of ILS), the lengths of internal branches of the subtrees approach zero accordingly. It follows that the probability distribution of a gene tree $\boldsymbol{{G}}$ for pseudoorthologs—converges to the coalescent distribution $P\left( {\boldsymbol{{G}}| \boldsymbol{{S}},\theta} \right)$ for orthologs (see Theorem 1 for the 4-taxon species tree and Theorem 2 for the n-taxon species tree in Supplementary Material E available on Dryad). Thus, when the level of ILS is high, concatenation methods are statistically inconsistent, whereas coalescent methods still perform reliably. If the internal branches of the species tree $\boldsymbol{{S}}$ are long (in coalescent units; i.e., a low level of ILS), the lengths of internal branches in the eight subtrees $\{\boldsymbol{{T}}_{\boldsymbol{{i}}},i = 1,\ldots,8\}$ should also be long. According to the coalescent theory, as the lengths of internal branches approach infinity, most of the gene trees $\boldsymbol{{G}}$ generated from the subtree $\boldsymbol{{T}}_{\boldsymbol{{i}}}$ are congruent with $\boldsymbol{{T}}_{\boldsymbol{{i}}}$ itself, that is,

$$
\begin{align*}
P\left( {\boldsymbol{{G}} = \boldsymbol{{T}}_{\boldsymbol{{i}}} | \boldsymbol{{T}}_{\boldsymbol{{i}}},\theta} \right) \to 1 \left( {i =
1, 2, \ldots, 8} \right).
\end{align*}$$

Consequently, the probability distribution of a gene tree $\boldsymbol{{G}}$ for pseudoorthologs converges to the probability distribution of the eight subtrees, that is,

$$
\begin{align*}
P\left( {\boldsymbol{{G}}| \boldsymbol{{S}},\theta,\lambda} \right) = \sum_{i = 1}^8
P\left( {\boldsymbol{{G}}| \boldsymbol{{T}}_{\boldsymbol{{i}}}, \theta} \right)P\left(
{\boldsymbol{{T}}_{\boldsymbol{{i}}} | S,\lambda} \right) \to P\left( {\boldsymbol{{T}}| \boldsymbol{{S}},\lambda} \right).
\end{align*}$$

In addition, as the lengths of the internal branches (Figs. S1a and S2a of the Supplementary material available on Dryad) in the species tree $\boldsymbol{{S}}$ approach infinity, the probability of a gene tree $\boldsymbol{{G}}$ that is identical to the species tree $\boldsymbol{{S}}$ converges to one. Since the species tree $\boldsymbol{{S}}$ is identical to the subtree $\boldsymbol{{T}}_{\bf 1}$, the coalescent distribution of gene trees $G$ for orthologs converges to a degenerated distribution $P\left( {\boldsymbol{{G}} = \boldsymbol{{T}}_{\bf 1} | \boldsymbol{{S}},\theta} \right) = 1$ as the lengths of internal branches of the species tree $\boldsymbol{{S}}$ approach infinity. Therefore, when the level of ILS is low, gene trees with pseudoorthologs are more variable than those with only orthologs. Specifically, the inclusion of pseudoorthologs artificially increases gene tree variation. Nevertheless, it can be shown that coalescent methods are statistically consistent in estimating the true species tree as the number of sampled genes increases (see Theorem 2 in Supplementary Material E available on Dryad). Furthermore, genes from one parental genome can be preferentially retained after hybridization (Thomas et al. 2006; Bird et al. 2018; Emery et al. 2018). In this case, when one of the two major subclades of paralogs is preferentially retained, that is, the pattern $\langle p_A,p_B,p_C,p_D > 0.5\rangle$ or $\langle p_A,p_B,p_C,p_D < 0.5\rangle$, the probability of the subtree $\boldsymbol{{T}}_{\bf 1}$ should be greater than the probability of any other subtree. Since the probability distribution of a gene tree $\boldsymbol{{G}}$ for pseudoorthologs is dominated by the coalescent distribution of a gene tree $\boldsymbol{{G}}$ generated from the species tree $\boldsymbol{{S}}$, coalescent methods consistently recover the species tree $\boldsymbol{{S}}$. In contrast, concatenation methods are inconsistent when the level of ILS is high (Kubatko and Degnan 2007).

### Investigating the Impact of Paralogs Using Simulated Data

**Figure 2.**
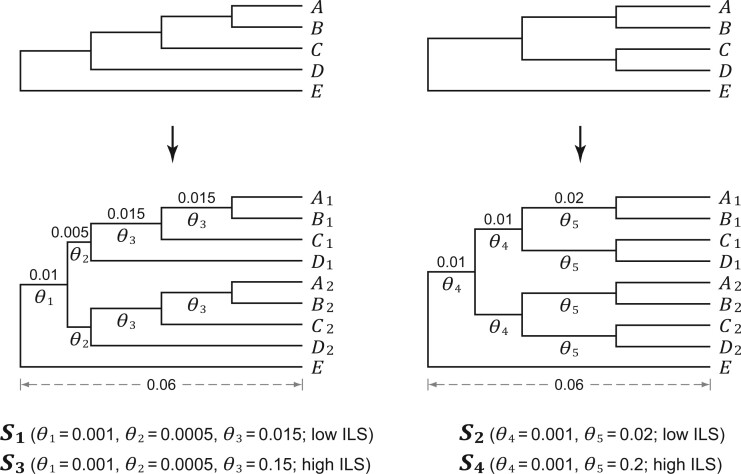
DNA simulations using 5-taxon species trees to investigate the impact of pseudoorthologs in the presence of ILS. Gene trees were simulated on ultrametric species trees $\boldsymbol{{S}}_{\bf 1}$ to $\boldsymbol{{S}}_{\bf 4}$ under the MSC (Rannala and Yang 2003), which were then utilized to simulate DNA sequences. The heights of these species trees are 0.06 (branch lengths are in mutation units and indicated above branches), and the population size parameter $\theta$ (shown below branches) is defined as $4\mu N_e$, where $N_e$ is the effective population size and $\mu$ is the average mutation rate per site per generation. In addition, a WGD was placed on the internal branch ancestral to ingroup species $A$ to $D$. For each gene, two alleles were sampled from each of the species $A$ to $D$, and one allele was sampled from the outgroup species $E$.

We additionally generated a differential loss of paralogs on numerous simulated data sets. DNA sequences were simulated on four 5-taxon species trees with two different topologies (i.e., pectinate species trees $\boldsymbol{{S}}_{\bf 1}$ and $\boldsymbol{{S}}_{\bf 3}$, and symmetrical species trees $\boldsymbol{{S}}_{\bf 2}$ and $\boldsymbol{{S}}_{\bf 4}$; Fig. 2) under the MSC (Rannala and Yang 2003). For each of the ultrametric species trees $\boldsymbol{{S}}_{\bf 1}$ to $\boldsymbol{{S}}_{\bf 4}$, species $E$ was designated as the outgroup and the height of the tree was held constant at 0.06 (lengths herein are reported in mutation units, i.e., the number of DNA substitutions per site). In addition, a WGD was placed on the internal branch ancestral to ingroup species $A$ to $D$. For each gene, two alleles were sampled from each of the species $A$ to $D$, and one allele was sampled from species $E$. The lengths of the internal branches following WGD were held constant (i.e., 0.015 for species trees $\boldsymbol{{S}}_{\bf 1}$ and $\boldsymbol{{S}}_{\bf 3}$, and 0.02 for species trees $\boldsymbol{{S}}_{\bf 2}$ and $\boldsymbol{{S}}_{\bf 4})$. To simulate varying levels of ILS, we applied different values of the population size parameter $\theta$ to those internal branches following WGD (i.e., 0.015 and 0.15 for species trees $\boldsymbol{{S}}_{\bf 1}$ and $\boldsymbol{{S}}_{\bf 3}$, respectively, and 0.02 and 0.2 for species trees $\boldsymbol{{S}}_{\bf 2}$ and $\boldsymbol{{S}}_{\bf 4}$, respectively; Fig. 2). The population size parameter $\theta$ is defined as $4\mu N_e$, where $N_e$ is the effective population size and $\mu$ is the average mutation rate per site per generation. To determine if our values of $\theta$ were comparable with empirical studies, we converted the lengths of the internal branches to coalescent units. To accomplish this, the branch lengths in mutation units must be divided by $\theta$. Here, we determine that the lengths of the internal branches in species trees $\boldsymbol{{S}}_{\bf 1}$ to $\boldsymbol{{S}}_{\bf 4}$ (i.e., 0.1, 1, and 10 coalescent units) are within the range of two well-studied examples: the branches in *Passerina* buntings (i.e., as short as 0.05 coalescent units) (Carling and Brumfield 2008; Degnan and Rosenberg 2006) and the two internal branches in the human–chimpanzee–gorilla–orangutan species tree (i.e., $\sim$1.2 and $\sim$4.2 coalescent units) (Rannala and Yang 2003; Degnan and Rosenberg 2006; Degnan and Rosenberg 2009). We simulated 50, 100, 200, 500, and 1000 gene trees on each of the species trees $\boldsymbol{{S}}_{\bf 1}$ to $\boldsymbol{{S}}_{\bf 4}$ using the R function *sim.coaltree.sp* as implemented in Phybase v1.5 (Liu and Yu 2010). Each gene tree was then utilized to simulate DNA sequences of 1000 base pairs using Seq-Gen v1.3.3 (Rambaut and Grassly 1997) with the JC69 model (Jukes and Cantor 1969). Each simulation was repeated 100 times, which resulted in a total of 500 data sets for each of the species trees $\boldsymbol{{S}}_{\bf 1} - \boldsymbol{{S}}_{\bf 4}$.

Next, we generated differential loss of paralogs on each simulated gene according to one of 14 patterns described below. In the simulation, the four-terminal lineages in the genealogical tree of the eight gene copies are removed according to the loss probabilities $\{p_x,x = A,B,C,D\}$ and then DNA sequences are simulated from the reduced tree, which is equivalent to simulating DNA sequences from the 8-taxon gene tree and then removing four sequences according to the loss probabilities $\{p_x,x = A,B,C,D\}$. The loss probabilities of the 14 patterns are resulted from various combinations of Scenarios 1–3. For the Pattern 1, that is, $\langle p_A = p_B = p_C = p_D = 1\rangle$, one of the two major subclades of paralogs was randomly selected for each gene, and all gene sequences belonging to this subclade were removed (Scenario 1). For the Pattern 2, that is, $\langle p_A = p_B = p_C = p_D = \frac{1}{2}\rangle$, one of a paralog pair was randomly selected and removed for each of the ingroup species $A$ to $D$ (Scenario 3). For the Pattern 3, that is, $\langle p_A = p_B = 1, \ \ p_C = p_D = 0\rangle$, one of the two major subclades of paralogs was first randomly selected and gene sequences of species $A$ and $B$ were removed from this subclade, and then gene sequences of species $C$ and $D$ were removed from another major subclade (Scenario 2: gene loss occurs in the internal branch leading to the ingroup species $A$ and $B)$. Similarly, paralog loss was generated for patterns $\langle p_A = p_C = 1, \ \ p_B = p_D = 0\rangle$ (Pattern 4) and $\langle p_A = p_D = 1, \ \ p_B = p_C = 0\rangle$ (Pattern 5). For the Pattern 6, that is, $\langle p_A = p_B = p_C = 1, \ \ p_D = 0\rangle$, one of the two major subclades of paralogs was first randomly selected and gene sequences of species $A$, $B$, and $C$ were removed from this subclade, and then the gene sequence of the species $D$ were removed from another major subclade. Similarly, loss of paralogs was generated for patterns $\langle p_A = p_B = p_D = 1, \ \ p_C = 0\rangle$ (Pattern 7) and $\langle p_A = p_C = p_D = 1, \ \ p_B = 0\rangle$ (Pattern 8). Finally, due to variation in selective constraints across lineages, duplicated genes can be convergently lost in different lineages (De Smet et al. 2013). For example, convergent loss of paralogs has shown to be about three times more frequent than reciprocal loss of paralogs in yeasts (Scannell et al. 2006; Scannell et al. 2007). For this reason, we simulated convergent loss of paralogs in the presence of ILS. For the Pattern 9, that is, $\langle p_A = p_B = 1, \ \ p_C = p_D = \frac{1}{2}\rangle$, one of an paralog pair was first randomly selected and removed within each gene for species $C$ and $D$, and then one of the two major subclades of paralogs was randomly selected and gene sequences of species $A$ and $B$ were removed from this subclade. Similarly, loss of paralogs was generated for patterns $\langle p_A = p_C = 1, \ \ p_B = p_D = \frac{1}{2}\rangle$ (Pattern 10) and $\langle p_A = p_D = 1, \ \ p_B = p_C = \frac{1}{2}\rangle$ (Pattern 11). For the Pattern 12, that is, $\langle p_A = p_B = p_C = 1, \ \ p_D = \frac{1}{2}\rangle$, one of an paralog pair was first randomly selected and removed within each gene for the species $D$, and then one of the two major subclades of paralogs was randomly selected and gene sequences of species $A$, $B$, and $C$ were removed from this subclade. Similarly, loss of paralogs was generated for patterns $\langle p_A = p_B = p_D = 1, \ \ p_C = \frac{1}{2}\rangle$ (Pattern 13) and $\langle p_A = p_C = p_D = 1, \ \ p_B = \frac{1}{2}\rangle$ (Pattern 14).

For single-copy genes generated according to each of the 14 patterns, species trees were inferred using alignment-based coalescent, gene-tree-based coalescent, and concatenation methods. The alignment-based coalescent analyses were conducted using BPP with the JC69 model. We ran each Markov chain Monte Carlo analysis for 100,000 generations, sampling trees and parameters every 10 generations. The consistency of stationary-phase likelihood values and estimated parameter values were determined using Tracer v1.7.1 (Rambaut et al. 2018). The estimated posterior distribution of the species tree was summarized from the last 1000 sampled posterior trees. Due to the computational burden, BPP was only run on the 50- and 100-gene data sets. For gene-tree-based coalescent analyses, gene trees were first inferred using RAxML v8.2.10 (Stamatakis 2014) with the GTRGAMMAX model (“-d -f o -m GTRGAMMAX -u”), and rooted with species $E$. These estimated gene trees were then utilized to construct species trees using ASTRAL v5.6.2, MP-EST v1.4, and the STAR method as implemented in Phybase (default settings were used for ASTRAL, MP-EST, and STAR). For concatenation analyses, species trees were inferred from concatenated gene sequences using optimality criteria maximum parsimony (CA-MP) and maximum likelihood (CA-ML). The best-scoring MP trees were inferred using PAUP* v.4.0a (Swofford 2002) with the branch-and-bound search, and the best-scoring ML trees were inferred using RAxML with the GTRGAMMAX model. Topological differences between inferred species trees and their true species tree were measured using the normalized Robinson–Foulds (RF) distance as implemented in RAxML (“-f r”). The normalized RF distance, or the RF distance (Kupczok et al. 2010), ranges between 0.0 and 1.0, and is calculated by dividing the RF metric (Robinson and Foulds 1981) by $2 \times \left( {n - 3} \right)$, where $n$ is the number of species. The mean RF distance was then calculated on the 100 data sets for each of the gene number categories (i.e., 50, 100, 200, 500, and 1000 genes).

### Examining the Impact of Pseudoorthologs Using Empirical Data

We further simulated loss of paralogs on two empirical data sets, which were newly assembled especially for this purpose from whole-genome sequencing data. Whole-genome studies have shown that there has been a relatively recent WGD in the ancestor of poplars and willows (i.e., the salicoid WGD; approximately 60–65 Ma) (Tuskan et al. 2006; Ma et al. 2013; Dai et al. 2014; Cai et al. 2019). Thus, we first assembled a data set using nuclear genome sequences of 12 species from the family Salicaceae *sensu lato* (i.e., *Idesia polycarpa*, *Populus alba*, *Populus deltoides*, *Populus euphratica*, *Populus ilicifolia*, *Populus pruinosa*, *Populus tremula*, *Populus tremuloides*, *Populus trichocarpa*, *Salix chaenomeloides*, *Salix purpurea*, and *Salix suchowensis*). Coding sequences were acquired from GigaDB (Sneddon et al. 2012), Phytozome (Goodstein et al. 2012), and the *Populus* Genome Integrative Explorer (PopGenIE; Sjödin et al. 2009). The establishment of sequence homology for amino acid sequences followed Xi et al. (2014). Each gene cluster was required to (i) include at least one sequence from $I$. *polycarpa* (for outgroup rooting), (ii) include two sequences from each of the 11 ingroup species, and (iii) include at least 100 amino acids for each sequence. For each gene cluster, amino acid sequences were aligned using the L-INS-i method as implemented in MAFFT v7.407 (Katoh and Standley 2013), and ambiguous sites were trimmed using trimAl v1.4.1 (Capella-Gutiérrez et al. 2009) with the heuristic automated method. DNA sequences were then aligned according to the corresponding amino acid alignments using PAL2NAL v14 (Suyama et al. 2006). Gene trees were reconstructed using RAxML with the GTRGAMMAX model. We examined the gene trees to verify that there are two major subclades and each subclade includes one sequence from each of 11 ingroup species. We removed the genes from analysis if the corresponding gene trees did not form two well-separated subclades. Orthologs were identified by the two subclades in the gene trees. Two subclades were rooted by the outgroup $I$. *polycarpa*. If the outgroup possessed only one sequence, two subclades were rooted with the same outgroup sequence.

Bootstrap support was estimated using a multilocus bootstrap approach (Seo 2008) with 100 replicates. These bootstrap gene trees were then utilized to construct species trees using ASTRAL, MP-EST, and STAR as described above. For CA-ML, the optimal partitioning schemes were first selected using the relaxed hierarchical clustering algorithm (Lanfear et al. 2014) as implemented in PartitionFinder v2.1.1 (Lanfear et al. 2017), and the best-scoring ML trees were then inferred using RAxML with the GTRGAMMAX model for each partition. For CA-MP, the best-scoring MP trees were inferred using PAUP* as described above. The bootstrap consensus trees were built using Phyutility v2.2.6 (Smith and Dunn 2008).

We simulated loss of paralogs according to one of the two patterns, that is, $\langle p_t = 1\rangle$ and $\langle p_t = \frac{1}{2}\rangle$. For the pattern $\langle p_t = 1\rangle$, one of the two major subclades of paralogs was randomly selected within each gene, and all gene sequences belonging to this subclade were removed; for the pattern $\langle p_t = \frac{1}{2}\rangle$, one of a paralog pair was randomly selected and removed for each of the 11 ingroup species. Species trees were then inferred from single-copy genes using gene-tree-based coalescent and concatenation methods as described above, and each simulation was repeated 100 times. The mean RF distances were calculated as described above to assess topological differences between the bootstrap consensus tree and those inferred from data sets with the simulated loss of paralogs.

We assembled a second empirical data set using nuclear genome sequences of nine budding yeasts (i.e., *Kluyveromyces africanus, Saccharomyces bayanus, Saccharomyces castellii, Saccharomyces cerevisiae, Saccharomyces dairenensis, Saccharomyces kudriavzevii, Saccharomyces mikatae, Saccharomyces naganishii*, and *Saccharomyces paradoxus*) from the family Saccharomycetaceae, in which a WGD was estimated to have occurred approximately 100–200 Ma (Morris and Drouin 2011; Wolfe et al. 2015). Amino acid sequences were acquired from the *Saccharomyces* Genome Database (Christie et al. 2004) and the Yeast Gene Order Browser (Byrne and Wolfe 2005). Sequence homology and sequence alignment were performed for amino acid sequences as described above, and only those gene clusters containing exactly two sequences from each of these nine budding yeasts were selected. The bootstrap consensus trees were built as described above. For gene-tree-based coalescent analyses, gene trees were first inferred using RAxML (“-d -f o -m PROTGAMMAAUTO -u –auto-prot=aicc”), and these estimated gene trees were then utilized to construct species trees using ASTRAL, MP-EST, and STAR. Loss of paralogs was simulated similarly according to the pattern $\langle p_t = 1\rangle$ or $\langle p_t = \frac{1}{2}\rangle$. Species trees were inferred from single-copy genes using gene-tree-based coalescent and concatenation methods as described above, and each simulation was repeated 100 times. The mean RF distances were calculated between the bootstrap consensus tree and those inferred from data sets with simulated loss of paralogs.

## Results and Discussion

### Simulated Data Sets

For each of the gene trees simulated on our 5-taxon species trees $\boldsymbol{{S}}_{\bf 1} - \boldsymbol{{S}}_{\bf 4}$ (Fig. 2), there were two major subclades of paralogs, and each subclade included exactly one sequence copy from each of the ingroup species $A - D$. The data sets simulated on species trees $\boldsymbol{{S}}_{\bf 1} - \boldsymbol{{S}}_{\bf 4}$ represent cases where speciation occurs shortly after WGD. Simulation analyses of species trees $\boldsymbol{{S}}_{\bf 1} - \boldsymbol{{S}}_{\bf 2}$ demonstrate that when $\theta$ was low (i.e., a low level of ILS), approximately 68$\%$ of the simulated gene trees (rooted with the outgroup species $E$) matched the topology of the species tree. When $\theta$ was high (i.e., a high level of ILS), only 2.5$\%$ and 4.3$\%$ of the simulated gene trees were congruent with species trees $\boldsymbol{{S}}_{\bf 3}$ and $\boldsymbol{{S}}_{\bf 4}$, respectively. Importantly, despite the highly discordant topologies among gene trees, the most probable gene tree still matched the species tree topology. Thus, species trees $\boldsymbol{{S}}_{\bf 1} - \boldsymbol{{S}}_{\bf 4}$ are not in the anomaly zone (Degnan and Rosenberg 2006). For data sets simulated on species trees $\boldsymbol{{S}}_{\bf 1} - \boldsymbol{{S}}_{\bf 4}$, loss of paralogs was generated according to one of the 14 patterns. For each of the simulated genes, one copy of a paralog pair was selected and removed based on the probability $p_t$ (where $t = A, B, C, D)$, resulting in single-copy genes with no missing data.

**Figure 3.**
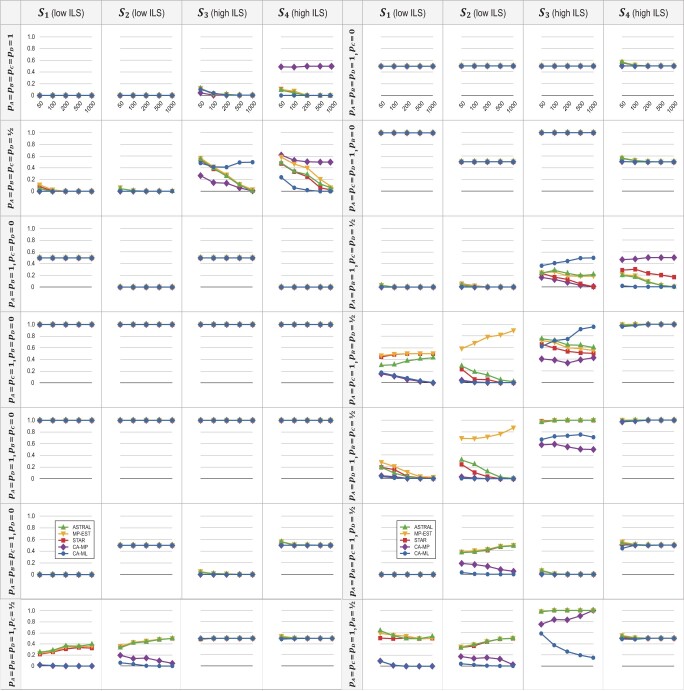
The mean RF distances between the true species tree and those inferred from data sets with simulated loss of paralogs. DNA sequences were simulated on species trees $\boldsymbol{{S}}_{\bf 1}$ to $\boldsymbol{{S}}_{\bf 4}$ (Fig. 2), and loss of paralogs was generated according to one of the 14 patterns as described in the Materials and Methods section. Species trees were then inferred from the 50-, 100-, 200-, 500-, and 1000-gene data sets using gene-tree-based coalescent (ASTRAL, MP-EST, and STAR) and concatenation (CA-MP and CA-ML) methods. The results for the Bayesian coalescent method BPP are in Figure S3 of the Supplementary material available on Dryad.

Since single-copy genes of the Pattern 1 consisted of only orthologs, all methods investigated in this study—alignment-based coalescent (BPP), gene-tree-based coalescent (ASTRAL, MP-EST, and STAR) and concatenation (CA-MP and CA-ML)—performed reliably when ILS was low (i.e., species trees $\boldsymbol{{S}}_{\bf 1}$ and $\boldsymbol{{S}}_{\bf 2} )$. The mean RF distances between the true species tree and those inferred by coalescent and concatenation methods were zero when the number of sampled genes was 50 (Fig. 3 and Fig. S3 of the Supplementary material available on Dryad). When ILS was high (i.e., species trees $\boldsymbol{{S}}_{\bf 3}$ and $\boldsymbol{{S}}_{\bf 4})$, both coalescent and concatenation methods (but not CA-MP, see below) still performed reliably as the number of sampled genes increased. Under these circumstances, despite the elevated gene tree estimation error resulting from short internal branches (the mean RF distances between simulated and inferred gene trees were 0.52 and 0.58 for species trees $\boldsymbol{{S}}_{\bf 3}$ and $\boldsymbol{{S}}_{\bf 4}$, respectively, versus only 0.077 and 0.099 for species trees $\boldsymbol{{S}}_{\bf 1}$ and $\boldsymbol{{S}}_{\bf 2}$, respectively), the mean RF distances between the true species tree and those inferred by coalescent methods were less than 0.075 as the number of sampled genes increased to 100. Similarly, the mean RF distance between the true species tree and those inferred by CA-ML was less than 0.035 as the number of sampled genes increased to 100. In contrast, the mean RF distance between the species tree $\boldsymbol{{S}}_{\bf 4}$ and those inferred by CA-MP increased to 0.50 as the number of sampled genes increased (Fig. 3). Here, although the topology of the species tree $\boldsymbol{{S}}_{\bf 4}$ was symmetrical, CA-MP consistently inferred one of the four pectinate trees (Fig. S4a of the Supplementary material available on Dryad) as the number of sampled genes increased to 1000. Therefore, a high level of ILS alone significantly increases the estimation error of species tree inference. Moreover, as the number of sampled genes increases, the performance of all methods is improved except for CA-MP, which converges to a tree that is different from the true species tree. These results corroborate the findings of previous studies (Roch and Steel 2015; Warnow 2015).

For Pattern 2, on average 87.5$\%$ of single-copy genes included pseudoorthologs. As compared with Pattern 1, the mean RF distances between two estimated gene trees increased from 0.21 to 0.73 for species trees $\boldsymbol{{S}}_{\bf 1}$ and $\boldsymbol{{S}}_{\bf 2}$, and increased from 0.77 to 0.79 for species trees $S_3$ and $S_4$. These results indicate that the inclusion of pseudoorthologs increases gene tree variation. Moreover, the internal branch lengths of the gene trees in Pattern 2 are longer than those in Pattern 1, indicating that the inclusion of pseudoorthologs can reduce the error rate for gene tree estimation. Compared with Pattern 1, the mean RF distances between simulated and inferred gene trees decreased from 0.077, 0.099, 0.52, and 0.58 to 0.058, 0.038, 0.28, and 0.23 for species trees $\boldsymbol{{S}}_{\bf 1}$ to $\boldsymbol{{S}}_{\bf 4}$, respectively. When ILS was low, the mean RF distances between the true species tree and those inferred by coalescent (BPP, Fig. S3 of the Supplementary material available on Dryad) and concatenation (Fig. 3) methods were less than 0.025 as the number of sampled genes increased to 100. When ILS was high, the accuracy of species tree estimation was adversely affected by the inclusion of pseudoorthologs. For example, the mean RF distances between the species tree $\boldsymbol{{S}}_{\bf 3}$ and those inferred by ASTRAL, MP-EST, and STAR were 0.39, 0.42, and 0.38, respectively, as the number of sampled genes was 100. Despite this adversity, the accuracy of coalescent methods was greatly improved by sampling more genes. For example, when the topology of the most frequently inferred gene tree was symmetrical, the mean RF distances between the pectinate species tree $\boldsymbol{{S}}_{\bf 3}$ and those inferred by ASTRAL, MP-EST, and STAR decreased to 0.010, 0.025, and 0.010, respectively, as the number of sampled genes increased to 1000. Thus, with the inclusion of pseudoorthologs, more loci are required to achieve the same level of accuracy. Under these circumstances, however, concatenation methods can produce inconsistent results. For example, even though the topology of the species tree $S_3$ was pectinate, CA-ML consistently inferred a symmetrical tree (Fig. S4b of the Supplementary material available on Dryad) as the number of sampled genes increased to 1000. Therefore, under these conditions, the performance of concatenation methods appears to be greatly compromised by the inclusion of pseudoorthologs.

For Patterns 3–8 (i.e., gene loss in the internal branches of the species tree), all single-copy genes included pseudoorthologs. For each pattern, the most likely subtree can be derived directly from the mathematical models described above. When the topology of the most likely subtree matched the true species tree (i.e., loss of paralogs was simulated according to Pattern 3 on species trees $\boldsymbol{{S}}_{\bf 2}$ and $\boldsymbol{{S}}_{\bf 4}$, and Pattern 6 on species trees $\boldsymbol{{S}}_{\bf 1}$ and $\boldsymbol{{S}}_{\bf 3}$), all methods accurately inferred the true species tree despite the degree of ILS (Fig. 3 and Fig. S3 of the Supplementary material available on Dryad). When the most likely subtree was incongruent with the true species tree, however, both coalescent and concatenation methods produced inconsistent results even for a low level of ILS. For example, when paralog loss was simulated according to Pattern 3 on the pectinate species trees $\boldsymbol{{S}}_{\bf 1}$ and $\boldsymbol{{S}}_{\bf 3}$, all methods consistently inferred a symmetrical tree (Fig. S4b of the Supplementary material available on Dryad), which was topologically identical with the most likely subtree. Therefore, these analyses corroborate results using the mathematical models described above, suggesting that both coalescent and concatenation methods are statistically inconsistent under these circumstances.

For Patterns 9–11, convergent loss of paralogs was constrained to occur in two of the four ingroup species $A$ to $D$, and on average three-quarters of single-copy genes consisted of pseudoorthologs. For Patterns 12–14, convergent loss of paralogs was constrained to occur in three of the four ingroup species $A$ to $D$, and on average one-half of single-copy genes consisted of pseudoorthologs. When ILS was low, alignment-based coalescent (BPP, Fig. S3 of the Supplementary material available on Dryad) and concatenation (Fig. 3) methods performed reliably as the number of sampled genes increased. Under these circumstances, however, gene-tree-based coalescent methods can produce inconsistent results. For example, when loss of paralogs was simulated according to Pattern 10, the most frequently inferred gene tree matched the topology of the species tree $\boldsymbol{{S}}_{\bf 1}$, but ASTRAL, MP-EST, and STAR consistently inferred an incorrect tree (Fig. S4c of the Supplementary material available on Dryad) as the number of sampled genes increased to 1000. Moreover, when convergent loss of paralogs was simulated on the symmetrical species tree $\boldsymbol{{S}}_{\bf 2}$, MP-EST unexpectedly inferred two pectinate trees (i.e., Fig. S4d of the Supplementary material available on Dryad for the Pattern 10, and Fig. S4e of the Supplementary material available on Dryad for the Pattern 11) as the number of sampled genes increased. The same results were obtained using MP-EST v2.0. As shown above, the inclusion of pseudoorthologs increases the lengths of the internal branches in the gene trees. Since gene-tree-based coalescent methods (e.g., ASTRAL, MP-EST, and STAR) estimate species trees based only on gene tree topologies, ignoring the branch-length information (Liu et al. 2015), under certain circumstances, it is challenging to accurately estimate the species trees in the presence of pseudoorthologs. On the other hand, BPP takes advantage of both topologies and branch lengths of gene trees in estimating species trees, and thus outperforms gene-tree-based coalescent methods when pseudoorthologs are included in single-copy genes. When the level of ILS was high, both gene-tree-based coalescent and concatenation methods were prone to produce inconsistent results (since BPP was only run on 50- and 100-gene data sets, its consistency is not discussed here). The exceptions were (i) when loss of paralogs was simulated according to Pattern 9, STAR and CA-MP accurately recovered the species tree $\boldsymbol{{S}}_{\bf 3}$ as the number of sampled genes increased to 1000, and ASTRAL, MP-EST, and CA-ML accurately estimated the species tree $\boldsymbol{{S}}_{\bf 4}$ as the number of sampled genes increased to 500 (Fig. 3); (ii) when loss of paralogs was simulated according to Pattern 12, all methods accurately estimated the species tree $\boldsymbol{{S}}_{\bf 3}$ as the number of sampled genes increased; (iii) when loss of paralogs was simulated according to Pattern 14, the mean RF distance between the species tree $\boldsymbol{{S}}_{\bf 3}$ and those inferred by CA-ML decreased to 0.15 as the number of sampled genes increased to 1000, which further decreased to 0.080 as the number of sampled genes increased to 2000.

### Empirical Data Sets

**Figure 4.**
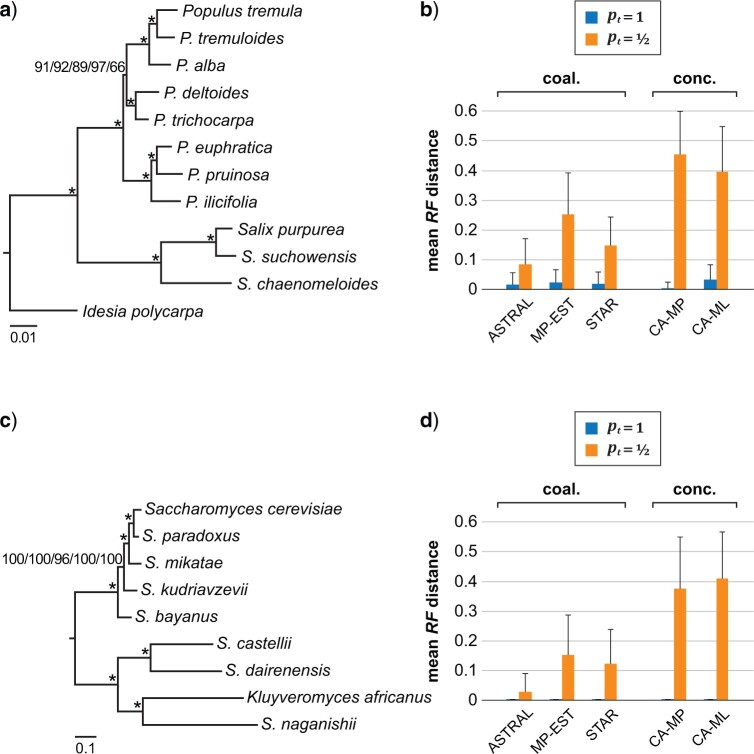
a) The bootstrap consensus tree of 12 Salicaceae species inferred from the 260-ortholog data set using gene-tree-based coalescent (ASTRAL, MP-EST, and STAR) and concatenation (CA-MP and CA-ML) methods. Branch lengths shown here (in mutation units) were estimated from concatenated DNA sequences using ML. BPs from ASTRAL/MP-EST/STAR/CA-MP/CA-ML are indicated for each internal branch, and an asterisk indicates 100 BPs in all analyses. b) The mean RF distances between the Salicaceae bootstrap consensus tree and those inferred from 130-gene data sets with simulated loss of paralogs. Loss of paralogs was generated on the 130-gene data set according to the pattern $\langle p_t = 1\rangle$ or $\langle p_t = \frac{1}{2}\rangle$ as described in the Materials and Methods section. Species trees were then inferred from single-copy genes using gene-tree-based coalescent and concatenation methods. The error bars represent one standard deviation from the mean. c) The bootstrap consensus tree of nine Saccharomycetaceae species inferred from the 210-gene data set using gene-tree-based coalescent and concatenation methods. Branch lengths shown here (in mutation units) were estimated from concatenated amino acid sequences using ML. BPs from ASTRAL/MP-EST/STAR/CA-MP/CA-ML are indicated for each internal branch, and an asterisk indicates 100 BPs in all analyses. d) The mean RF distances between the Saccharomycetaceae bootstrap consensus tree and those inferred from 105-gene data sets with simulated loss of paralogs. Loss of paralogs was generated on the 105-gene data set according to the pattern $\langle p_t = 1\rangle$ or $\langle p_t = \frac{1}{2}\rangle$. Species trees were then inferred from single-copy genes using gene-tree-based coalescent (ASTRAL, MP-EST, and STAR) and concatenation (CA-MP and CA-ML) methods. The error bars represent one standard deviation from the mean.

Our first empirical data set consisted of 130 genes from 12 Salicaceae species. The average number of nucleotide sites for each gene was 881 (ranging from 318 to 2547). Within each of the 130 genes, there were two major subclades of paralogs, and each subclade included one sequence from each of the 11 *Populus* and *Salix* species. For bootstrap analyses, the two major subclades of paralogs were divided into two gene clusters of orthologs, resulting in data set including 260-orthologs. Here, the mean RF distance between two gene trees was 0.30. Our gene-tree-based coalescent and concatenation analyses of the 260-ortholog data set resulted in a species tree with all clades receiving 100 bootstrap percentage (BP) support, except the one containing *P. alba, P. deltoides, P. trichocarpa, P. tremula*, and *P. tremuloides* (i.e., 91, 92, 89, 97, and 66 BP for ASTRAL, MP-EST, STAR, CA-MP, and CA-ML respectively; Fig. 4a).

Paralog loss was simulated on the 130-gene data set according to the pattern $\langle p_t = 1\rangle$ or $\langle p_t = \frac{1}{2}\rangle$, resulting in 130 single-copy genes with no missing data. Our phylogenomic analyses generally corroborated results using simulated data described above. As expected, for the pattern $\langle p_t = 1\rangle$, there was minimal effect on the accuracy of species tree estimation. For gene-tree-based coalescent and concatenation analyses, the mean RF distances between the bootstrap consensus tree and those inferred from data sets with simulated paralog loss ranged from 0.0044 to 0.033 (Fig. 4b). For the pattern $\langle p_t = \frac{1}{2}\rangle$, nearly all single-copy genes (99.9$\%$) included pseudoorthologs. Here, the accuracy of species tree estimation was adversely affected, and concatenation methods were more severely impacted. The mean RF distances between the bootstrap consensus tree and those inferred by ASTRAL, MP-EST, and STAR were 0.086, 0.25, and 0.15, respectively, while the mean RF distances between the bootstrap consensus tree and those inferred by CA-MP and CA-ML were 0.46 and 0.40, respectively (Fig. 4b).

Our second empirical data set consisted of 105 genes from nine post-WGD budding yeasts. The average number of amino acid sites for each gene was 623 (ranging from 145 to 2445). Similarly, within each of the 105 genes, there were two major subclades of paralogs, and each subclade included exactly one sequence from each of the nine yeasts. For bootstrap analyses, the two major subclades of paralogs were similarly divided into two gene clusters, resulting in a 210-gene data set. Here, the mean RF distance between two gene trees was 0.32. Our gene-tree-based coalescent and concatenation analyses of the 210-gene data set resulted in a highly supported (i.e., $ \ge$ 96 BP) species tree (Fig. 4c).

Loss of paralogs was simulated similarly on the 105-gene data set according to the pattern $\langle p_t = 1\rangle$ or $\langle p_t = \frac{1}{2}\rangle$, resulting in 105 single-copy genes with no missing data. The pattern $\langle p_t = 1\rangle$ had no effect on the accuracy of species tree estimation—species relationships inferred by the gene-tree-based coalescent and concatenation methods were identical to those inferred from the 210-gene data set (thus the mean RF distances were zero; Fig. 4d). In contrast, the pattern $\langle p_t = \frac{1}{2}\rangle$ substantially deteriorated the accuracy of species tree estimation, especially for concatenation methods. Here, the mean RF distances between the bootstrap consensus tree and those inferred by ASTRAL, MP-EST, and STAR were 0.028, 0.15, 0.12, respectively, while the mean RF distances between the bootstrap consensus tree and those inferred by CA-MP and CA-ML were 0.38 and 0.41, respectively (Fig. 4d). Therefore, our analyses of these two empirical data sets demonstrate that if both copies of a paralog pair in post-WGD species are equally likely to be lost, the inclusion of pseudoorthologs adversely affects the accuracy of species tree estimation, especially when analyzed with concatenation methods.

## Conclusions

The majority of phylogenomic inference programs prioritize single-copy orthologous genes as input molecular markers for most accurate inference. When speciation occurs shortly after WGD, subsequent loss of paralogs can lead to the spurious inclusion of pseudoorthologs in single-copy genes. Our analyses demonstrate that the inclusion of pseudoorthologs can detrimentally influence species tree estimation, but that the adverse effect depends on the pattern of gene loss following WGD. When gene loss occurs at the root of the species tree, the remaining copy evolves following the species tree, resulting in orthologous genes that can be used for accurately reconstructing the species trees under the MSC. However, gene loss among the internal branches (i.e., ancestral populations) of the species tree causes joint loss of gene copies for all descendant species, producing incongruent paralog trees (i.e., the eight subtrees in Supplementary Material B available on Dryad). The inclusion of pseudoorthologs in these cases can mislead both concatenation and coalescent methods in species tree estimation. Therefore, pseudoorthologs become especially problematic in the presence of gene loss among the ancestral lineages of the species tree, where both coalescent and concatenation methods are prone to produce inconsistent results. Since the probability of gene loss among ancestral lineages depends on the length of the internal branches, extreme caution is required when analyzing data consisting of post-WGD single-copy genes for a species tree with long internal branches due to incomplete species sampling, massive extinction, or incomplete species sampling.

Our results will hopefully lead to the refinement and further development of models and methods that are more robust to the adverse effect of pseudoorthologs on species tree estimation. For example, a mathematical model can simultaneously reconstruct gene and species trees in the presence of gene duplication and loss (Boussau et al. 2013), and several new methods have been developed to deal with multicopy genes while modeling gene duplication and loss (Zhang et al. 2020; Molloy and Warnow 2020; Morel et al. 2021). Interestingly, a recent study indicates that treating all copies (paralogs and orthologs) within a gene family as multiple alleles from each species can provide very accurate results (Du et al. 2019). Besides WGD, pseudoorthologs can additionally originate from single-gene duplications, which include tandem, proximal, DNA-based transposed, retrotransposed, and dispersed duplications (Wang et al. 2012). As a result, the adverse effect of pseudoorthologs may be more profound than anticipated and should be more carefully considered and properly accommodated during phylogenomic analyses.

If gene loss occurs at random among the terminal branches of the species tree (Scenario 3), the inferred majority paralog tree remains congruent with the species tree, which is consistent with the previous results that the probability of the discordant paralog tree is lower than that of the concordant paralog tree (Smith and Hahn 2022). For Scenario 3, our analysis indicates that coalescent methods are statistically consistent and perform more reliably than concatenation methods in species tree estimation as the number of genes increases. Moreover, for multicopy genes, randomly selecting a copy per species is equivalent to random gene loss occurring among the terminal branches of the species tree, suggesting that coalescent-based species tree inference is robust to the presence of paralogs resulting from multicopy genes (Yan et al. 2022). In our analysis, pseudoorthologs are problematic when filtering only for single-copy genes in phylogenomic data sets generated from genomes and transcriptomes in groups with recurrent WGD. Pruning orthologs (Yang and Smith 2014) or even randomly selecting a copy from multi-copy genes can avoid most of those pseudoorthologs.

## Supplementary material

Data available from the Dryad Digital Repository: http://dx.doi.org/10.5061/dryad.prr4xgxmr.
